# Patients with Periprosthetic Femoral Hip Fractures are Commonly Classified as Having Osteoporosis Based on DXA Measurements

**DOI:** 10.1007/s00223-024-01237-w

**Published:** 2024-06-04

**Authors:** Jacob Ritter, Assil-Ramin Alimy, Alexander Simon, Jan Hubert, Christian Ries, Tim Rolvien, Frank Timo Beil

**Affiliations:** Division of Orthopaedics, Department of Trauma and Orthopaedic Surgery, University Medical Center Hamburg-Eppendorf, Martinistraße 52, 20246 Hamburg, Germany

**Keywords:** Total hip arthroplasty, Osteoporosis, Periprosthetic fracture, Dual-energy X-ray absorptiometry

## Abstract

Periprosthetic femoral hip fractures are subject to an increasing incidence and are often considered to be related to osteoporosis. However, there are no available studies that have determined the frequency of osteoporosis in affected patients using gold standard dual-energy X-ray absorptiometry (DXA). In this retrospective comparative study, we analyzed the DXA results of 40 patients with periprosthetic femoral hip fractures who were treated surgically in our department. DXA measurements were performed at the total hip and the lumbar spine to determine bone mineral density T-scores. Data were compared to two age-, sex-, and BMI-matched control groups in which patients underwent DXA prior to aseptic revision surgery for other causes or primary THA (consisting of 40 patients each). The mean T-score in the periprosthetic fracture cohort was significantly lower (− 1.78 ± 1.78) than that of the aseptic revision (− 0.65 ± 1.58, mean difference − 1.13 [95% CI − 1.88 to − 0.37]; *p* = 0.001) and the primary THA cohort (− 0.77 ± 1.34, mean difference − 1.01 [95% CI − 1.77 to − 0.26]; *p* = 0.005). Accordingly, osteoporosis was detected more frequently (45%) in the fracture cohort compared to patients undergoing aseptic revision (12.5%) and primary THA (10%). In conclusion, almost half of the patients with periprosthetic femoral hip fractures have osteoporosis according to DXA measurements. A regular assessment of bone health in THA enables identification of patients with osteoporosis who likely benefit from initiation of osteoporosis medication and cemented stem fixation.

**Supplementary Information:**

The online version contains supplementary material available at 10.1007/s00223-024-01237-w.

## Introduction

As the population ages and the demand to retain mobility and quality of life with advancing age grows, the number of endoprosthetic procedures is increasing worldwide. This increase, however, has led to a rise in complications from these surgical procedures. A serious complication after total hip arthroplasty (THA), a highly successful surgical procedure to treat end-stage hip osteoarthritis (OA), is periprosthetic femoral hip fracture (PPF) [1]. PPF is defined as a fracture around the femoral stem, which can occur either intra-or postoperatively [2]. PPF is considered the third most common cause of revision surgery after primary THA [3]. Patients with PPF often face worse functional outcomes compared to those undergoing primary THA or revision for aseptic loosening [4]. Risk factors for PPF include advanced age, female sex, infections, rheumatoid arthritis, and uncemented stem fixation [2].

Another assumed risk factor for PPF is poor bone quality (i.e., low BMD or osteoporosis), which has led to these fractures being referred to as an ‘‘osteoporosis crisis’’ or ‘‘osteoporosis epidemic’’ [5, 6]. Higher age and female sex have previously been described as potential risk factors for PPF [7, 8], pointing to an overlap with patients at risk for osteoporosis. The World Health Organization (WHO) defines the diagnosis of osteoporosis based on dual-energy X-ray absorptiometry (DXA) measurements, as a bone mineral density (BMD) standard deviation (i.e., T-score) of − 2.5 or below compared to a reference cohort of young, skeletally healthy adults. If the T-score is between − 1.0 and − 2.5, the diagnosis of osteopenia is made. Osteoporosis and osteopenia are common comorbidities in patients scheduled for THA [9], which are often underrecognized and undertreated [10]. The International Society for Clinical Densitometry (ISCD) has provided recommendations for assessing BMD by DXA preoperatively in high-risk patients scheduled for THA, but these are hardly implemented in daily clinical practice [11].

While osteoporosis is a well-established risk factor for fragility fractures of the femur and spine, there is a notable paucity of studies that have previously assessed the role of low BMD in the context of PPF. This also raises a critical question: How common is osteoporosis in patients with PPF? Therefore, the aim of this study was to examine the frequency of osteoporosis in patients with PPF based on DXA measurements. Further aims of this study were to compare DXA T-scores with two control cohorts from the hip arthroplasty spectrum as well as within the PPF cohort based on clinical constellations such as intra- vs. postoperative fracture and cemented vs. uncemented stem fixation. Identification of osteoporosis appears clinically highly relevant, given the assumption that optimizing bone health and, more specifically, anti-osteoporosis medications would substantially reduce the risk of PPF. Therefore, we also aim to raise the awareness, encourage the evaluation of preoperative BMD in high-risk patients, and adapt surgical concepts based on BMD outcomes.

## Materials and Methods

### Study Design and Patient Cohorts

**Fig. 1:**
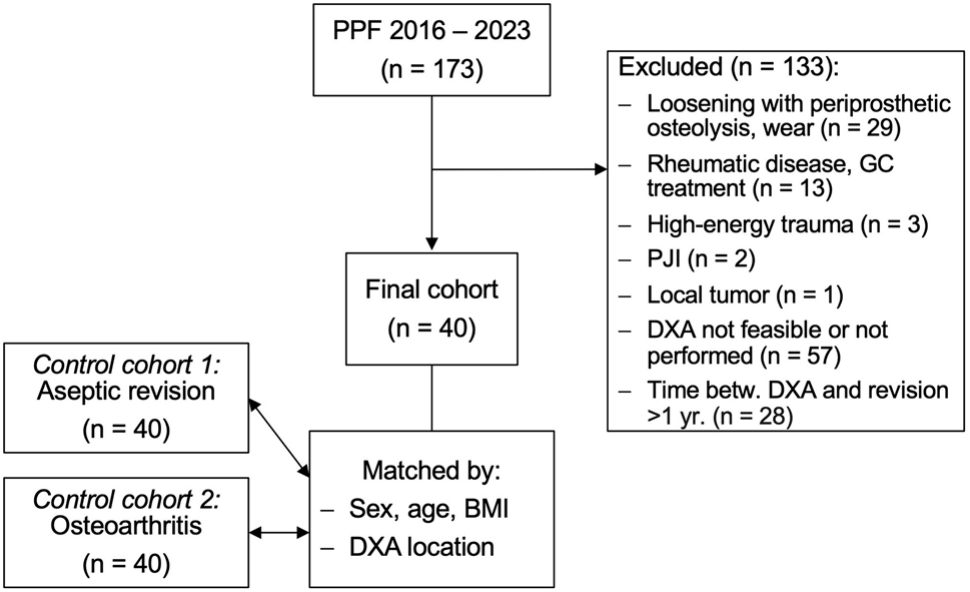
Flowchart. Retrospective identification of the study population, consisting of patients with periprosthetic femoral hip fracture (PPF), exclusion of other potential causes, and available dual-energy X-ray absorptiometry (DXA) measurement within one year before or after the revision surgery. *BMI* body mass index, *GC* glucocorticoid, *PJI* periprosthetic joint infection, *yr.* year

Between January 2016 and December 2023, we screened 173 patients treated surgically for PPF at our institution. Of those, 133 patients were excluded because of loosening, periprosthetic osteolysis, or wear (29 patients), rheumatic diseases and/or glucocorticoid treatment (13), high-energy trauma (three), periprosthetic joint infection (two), a local tumor (one), a DXA measurement was not performed due to implants or advanced degeneration of both the contralateral hip and the lumbar spine (57), or a period of more than one year between fracture and DXA measurement (28). Consequently, a total of 40 patients remained eligible for analysis in this retrospective, comparative study (Fig. 1). Most fractures occurred after a fall (38/40, 95%), and only two fractures occurred postoperatively without trauma (i.e., spontaneously).

Demographic data, including age, sex, and body mass index (BMI), were assessed. Furthermore, detailed medical history was obtained in all patients. Factors evaluated included the time between primary THA and fracture (i.e., survival), the mode of fixation (cemented vs. uncemented), the fracture type according to the Vancouver classification, and the timing of fracture occurrence (intra-or postoperatively). Treatment with vitamin D and antiresorptive or osteoanabolic drugs was also analyzed, if taken prior to admission to our department.

The PPF cohort was compared with two control groups: patients undergoing aseptic revision surgery (AR—control cohort 1) and patients undergoing primary THA for osteoarthritis (OA—control cohort 2). Case–control matching based on demographic data (age, sex, and BMI) was performed. Each of the three cohorts consisted of 40 patients who did not differ in age (PPF vs. AR: 72.5 ± 11.1 vs. 72.5 ± 9.3 years, mean difference 0.0 years [95% CI − 2.08 to 2.08]; *p* > 0.99; PPF vs. OA: 72.5 ± 11.1 vs. 71.6 ± 10.3 years, mean difference 0.91 years [95% CI − 1.16 to 2.99]; *p* = 0.534), sex ratio (each 26 females and 14 males; *p* > 0.99), BMI (PPF vs. AR: 26.4 ± 4.0 vs. 27.2 ± 4.5 kg/m^2^, mean difference − 0.79 kg/m^2^ [95% CI − 1.89 to 0.29]; *p* = 0.217; PPF vs. OA: 26.4 ± 4.0 vs. 27.1 ± 3.7 kg/m^2^, mean difference − 0.87 kg/m^2^ [95% CI − 1.94 to 0.21]; *p* = 0.150) and DXA measurement site (each 35 lumbar spine and 26 femurs; *p* > 0.99). The implant survival time until revision surgery was significantly shorter in the PPF cohort compared to the AR control cohort (6.6 ± 9.5 vs. 9.9 ± 9.9 years; mean difference 3.3 years [95% CI 0.25 to 5.7]; *p* = 0.005).

### Dual-Energy X-ray Absorptiometry

Areal bone mineral density (aBMD) was assessed by dual-energy X-ray absorptiometry (DXA, Lunar Prodigy, enCore 2005, version 9.15.010, GE Healthcare; Madison, WI, USA) performed at both proximal femora (total hip) and the lumbar spine (L1-L4). All DXA scans were reviewed by the study team to avoid errors in acquisition and interpretation. Total hip and lumbar spine T-scores, representing the BMD standard deviations in relation to 20-to 40-year-old sex-matched healthy adults, and Z-scores, representing the BMD standard deviations in relation to age-and sex-matched healthy individuals, were generated according to national guidelines [12]. Manufacturer-specific reference databases of a German cohort were used to calculate total hip and lumbar spine T-scores and Z-scores. In accordance with the WHO criteria, a diagnosis of osteoporosis was made if the lowest T-score was ≤ − 2.5, or a diagnosis of osteopenia was made if the lowest T-score was between − 1.0 and − 2.5 [13]. A T-score of ≥ − 1.0 was considered a normal BMD. If the proximal femur could not be measured due to the presence of a prosthesis, the other side or the lumbar spine (in the case of bilateral THA) was included in the evaluation. In the lumbar spine, degenerative vertebral bodies were excluded, whereby at least two adjacent vertebral bodies were used to calculate the T-and Z-scores. In 5 of 40 patients, the lumbar spine measurements were fully excluded due to severe degenerative changes.

Precise matching of the DXA measurement site was performed between the three cohorts (Suppl. Figure 1). For instance, in the case of a PPF in a patient with a right-sided THA and postoperative DXA measurement of the left femur and lumbar spine, a matched patient from either of the control cohorts also had to have right-sided OA or THA, with DXA measurement similarly taken on the left proximal femur and lumbar spine.

### Statistical Analysis

Statistical analysis of the data and visualization of the results were performed using Prism version 10.1.1 (GraphPad Software Inc., La Jolla, CA, USA) and Statistical Product and Service Solutions (SPSS) Statistics version 29.0 (IBM, Armonk, NY, USA). After confirming normal distribution, we used an unpaired or paired two-tailed *t*-test for comparison of two groups, or the Mann–Whitney U-test for comparison of two groups with non-normally distributed data. The comparison of more than two groups was performed either with one-way analysis of variance (ANOVA) with Tukey’s post hoc analysis for normally distributed data or with the Kruskal–Wallis test with Dunn’s post hoc analysis for non-normally distributed data. Comparison between two categorical variables was performed using the Chi-squared test. The level of significance was defined as *p* < 0.05. Exact p-values are reported unless *p* < 0.001. Data are displayed as mean ± standard deviation (SD) or as boxplot with median, interquartile range, minimum, and maximum, as well as all plotted data points.

## Results

### Case Study

**Fig. 2:**
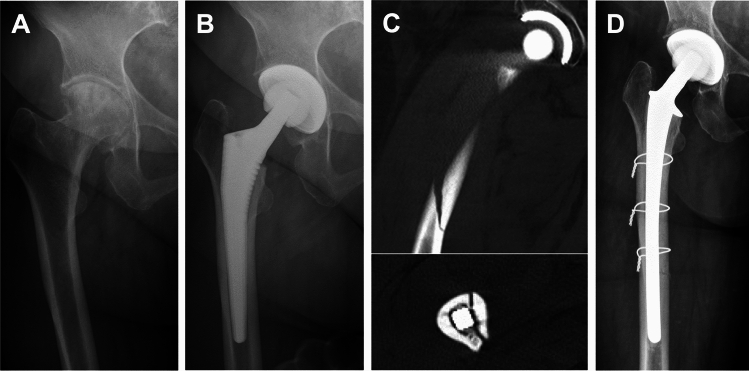
Exemplary case study of a 65-year-old woman with osteoporosis and a postoperative periprosthetic femoral hip fracture (PPF). **A** Preoperative radiograph showing end-stage OA and Dorr C type femur. **B** Postoperative radiograph after uncemented THA without evidence of a fracture, **C** computed tomography (coronal and axial view) showing a PPF (Vancouver type B2), **D** radiograph after stem revision

A 65-year-old woman with end-stage OA of the right hip underwent uncemented THA at our department (Fig. 2 A, B). DXA had been performed preoperatively due to the presence of bone-related risk factors (age, family history, hyperthyroidism). With a T-score of − 2.7, the diagnosis of osteoporosis was made. While no bone-specific medication was initiated prior to THA, vitamin D supplementation had been initiated in the past. One month after THA, the patient complained of progressive pain in her right thigh following a torsional trauma during rehabilitation. The subsequent radiological examination revealed a PPF (Vancouver type B2) (Fig. 2 C), treated by open reduction, cerclage wire fixation, and cemented stem revision (Fig. 2 D).

### Frequency of Osteoporosis and Related Treatments in the PPF Cohort

We observed fewer patients with normal BMD in the PPF cohort compared to both control cohorts (PPF vs. AR: 32.5 vs. 65%; *p* = 0.007; PPF vs. OA: 32.5 vs. 60%; *p* = 0.04) (Table 1). Accordingly, we detected osteoporosis in 45% of patients with PPF and only 12.5% and 10% in the aseptic revision and primary THA control cohorts, respectively (PPF vs. AR: *p* = 0.003; PPF vs. OA: *p* < 0.001). In accordance with the higher frequency of osteoporosis in the PPF group, more patients were treated with vitamin D (PPF vs. AR: 55 vs. 37.5%; *p* = 0.18; PPF vs. OA: 55 vs. 17.5%; *p* < 0.001) or antiresorptive medication (PPF vs. AR: 22.5 vs. 2.5%; *p* = 0.01) prior to admission to our institution (Table 1).

**Table 1:** Comparison of demographic, clinical, and radiographic parameters between the periprosthetic fracture cohort and the two control cohorts

Parameters mean (± SD)	PPF *n* = 40	AR *n* = 40	OA *n* = 40	*p*-value (PPF vs. AR)	*p*-value (PPF vs. OA)
Age (yr.)	72.5 (± 11.1)	72.5 (± 9.3)	71.6 (± 10.3)	> 0.99	0.534
Sex ratio (f/m)	26/14	26/14	26/14	> 0.99	> 0.99
BMI (kg/m^2^)	26.4 (± 4.0)	27.2 (± 4.5)	27.1 (± 3.7)	0.218	0.150
Time in situ (yr.)	6.6 (± 9.5)	9.9 (± 9.9)	–	**0.005**	–
Lowest T-score	− 1.78 (± 1.78)	− 0.65 (± 1.58)	− 0.77 (± 1.34)	**0.001**	**0.005**
Lowest Z-score	− 0.62 (± 1.69)	0.36 (± 1.52)	0.36 (± 1.29)	**0.004**	**0.004**
Normal BMD (%)	13/40 (32.5)	26/40 (65)	23/40 (57.5)	**0.007**	**0.040**
Osteopenia (%)	9/40 (22.5)	9/40 (22.5)	13/40 (32.5)	> 0.99	0.450
Osteoporosis (%)	18/40 (45)	5/40 (12.5)	4/40 (10)	**0.003**	** < 0.001**
Vitamin D (%)	22/40 (55)	15/40 (37.5)	7/40 (17.5)	0.180	** < 0.001**
BP/Dmab (%)	9/40 (22.5)	1/40 (2.5)	–	**0.010**	–

Bold indicates significant differences

*PPF* periprosthetic fracture, *AR* aseptic revision, *OA* osteoarthritis, *yr.* years, *f* female, *m* male, *BMI* body mass index, *BMD* bone mineral density, *BP* bisphosphonates, *Dmab* denosumab

### Comparison of DXA Outcomes Between the PPF Cohort and Both Control Cohorts

**Fig. 3:**
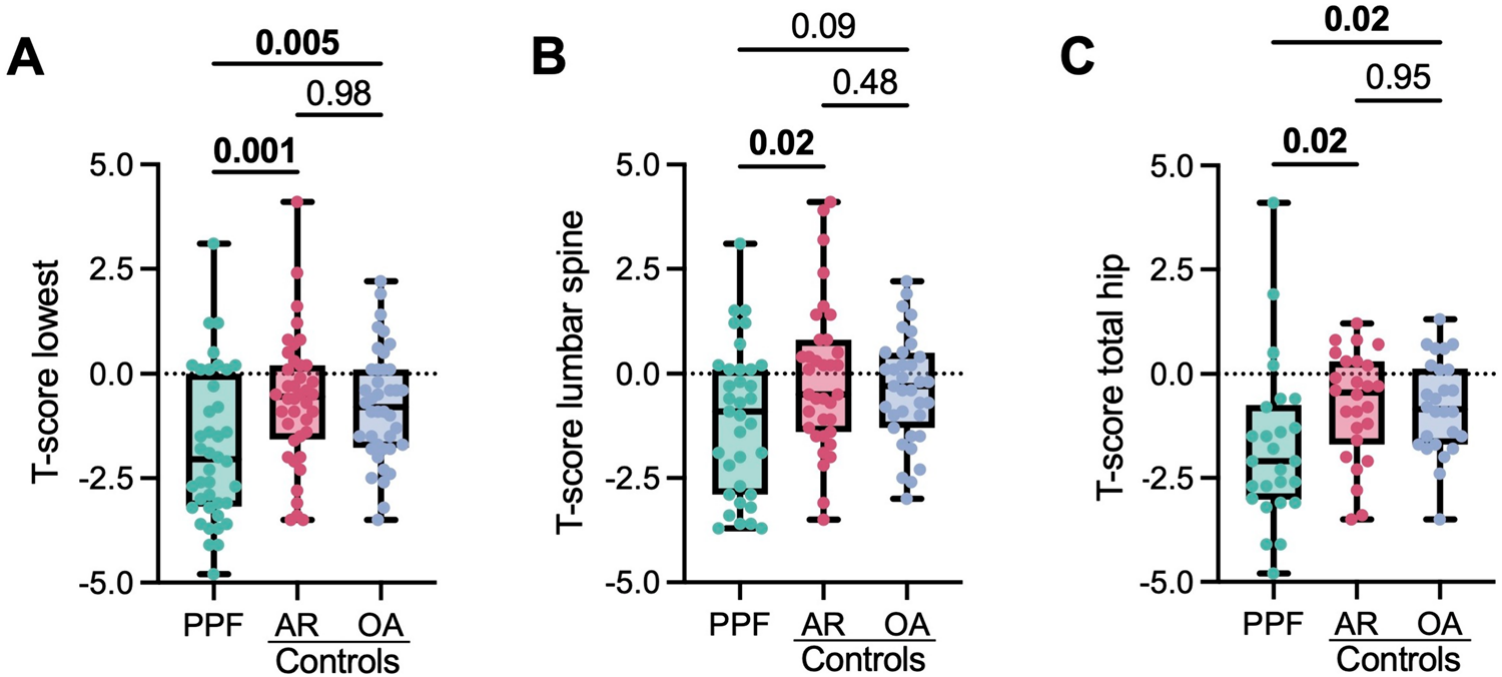
Comparison of BMD T-scores assessed by DXA between the periprosthetic fracture (PPF) cohort and both control cohorts. Comparison of T-scores when evaluating the **A** lowest T-score of any measurement site, **B** lumbar spine, and **C** total hip. Bold indicates significant differences. *AR* aseptic revision, *OA* osteoarthritis

When considering the lowest value of any DXA measurement site, the T-score in the PPF cohort was significantly lower than that of the two control cohorts (PPF vs. AR: − 1.78 ± 1.78 vs. 0.65 ± 1.58, mean difference − 1.13 [95% CI − 1.88 to − 0.37]; *p* = 0.001; PPF vs. OA: − 1.78 ± 1.78 vs. 0.77 ± 1.34, mean difference − 1.01 [95% CI − 1.77 to − 0.26]; *p* = 0.005) (Fig. 3 A). Similarly, the Z-score was significantly lower in the PPF cohort (PPF vs. AR: (− 0.62 ± 1.69 vs. 0.36 ± 1.52, mean difference − 0.98 [95% CI − 1.69 to − 0.26]; *p* = 0.004; PPF vs. OA: (− 0.62 ± 1.69 vs. 0.36 ± 1.29, mean difference − 0.98 [95% CI − 1.70 to − 0.27]; *p* = 0.004) (Suppl. Figure 2). To demonstrate the independence of DXA values from measurement site, we also compared the T-and Z-scores individually for each site. In the proximal femur, lower T-and Z-scores were detected in the PPF cohort compared to both control groups (Table 2, Fig. 3 B, C; Suppl. Figure 2). In the lumbar spine, the comparison between the PPF cohort and the OA cohort marginally failed to reach the significance level, while the difference between PPF and AR was also significant. It was also evident that the two control cohorts showed no differences in T-and Z-scores in all evaluations.

**Table 2:** Comparison of mean T-and Z-scores stratified by DXA measurement site between the periprosthetic fracture cohort and the two control cohorts

DXA measurement site mean (± SD)	PPF *n* = 40	AR *n* = 40	OA *n* = 40	*p*-value (PPF vs. AR)	*p*-value (PPF vs. OA)
Lumbar spine (*n*)	35	35	35		
T-score	− 1.09 (± 1.78)	− 0.12 (± 1.77)	− 0.37 (± 1.29)	**0.022**	0.087
Z-score	− 0.03 (± 1.78)	0.80 (± 1.70)	0.54 (± 1.32)	**0.049**	0.190
Total hip (*n*)	26	26	26		
T-score	− 1.74 (± 1.92)	− 0.76 (± 1.30)	− 0.79 (± 1.13)	**0.023**	**0.023**
Z-score	− 0.61 (± 1.70)	0.19 (± 1.37)	0.25 (± 0.94)	**0.035**	**0.029**

Bold indicates significant differences

*DXA* dual-energy X-ray absorptiometry, *PPF* periprosthetic fracture, *AR* aseptic revision, *OA* osteoarthritis

### DXA Values Within the PPF Cohort According to Clinical Constellations

**Fig. 4:**
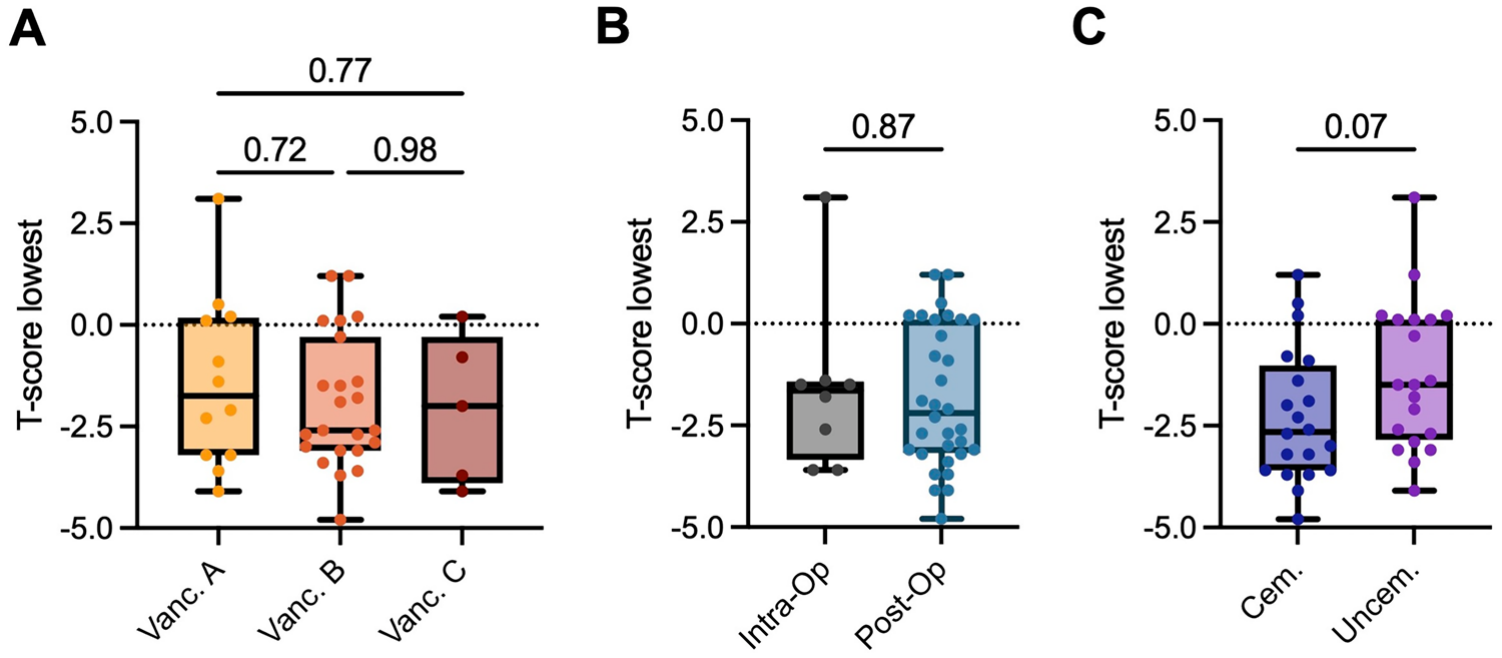
Comparison of BMD T-scores according to different clinical constellations. **A** Comparison of T-scores (lowest of any measurement site) between different types of PPF according to the Vancouver classification, **B** between intraoperative and postoperative fractures, and **C** between patients undergoing cemented vs. uncemented fixation

The BMD T-and Z-scores were also analyzed and compared within the PPF cohort based on clinical constellations. We observed 12, 23, and 5 Vancouver A, B, and C fractures, respectively. No differences were found for the comparison of T-or Z-scores between fracture types according to the Vancouver classification (Fig. 4 A, Suppl. Figure 3). Furthermore, no differences were observed between patients suffering from an intraoperative (*n* = 8) vs. postoperative fracture (*n* = 32) (T-score, − 1.61 ± 2.11 vs. − 1.82 ± 1.73, mean difference − 0.21 [95% CI − 1.6 to 1.6]; *p* = 0.87) (Fig. 4 B, Suppl. Figure 3). Notably, of the eight intraoperative fractures, six (75%) occurred with uncemented stem fixation, and three and five were classified as Vancouver A and B, respectively. No significant differences in DXA outcomes were also observed regarding the comparison between cemented (*n* = 20) and uncemented (*n* = 20) stem fixation (T-score, − 2.28 ± 1.63 vs. − 1.28 ± 1.82, mean difference − 1.0 [95% CI − 0.10 to 2.11]; *p* = 0.07) (Fig. 4 C, Suppl. Figure 3), although a trend toward lower T-scores with cemented fixation was observed.

## Discussion

PPF is a serious complication of THA, with an incidence on the rise [2, 10]. Previous studies have suggested that PPF could be of osteoporotic origin, as osteoporosis-related factors (age, female sex) were frequently observed in affected patients [7, 8]. However, while osteoporosis is typically considered as a contributing cause [14], no cross-sectional study has systematically investigated the frequency of osteoporosis in affected patients and compared DXA parameters with adequately matched control groups. Therefore, in this study, DXA outcomes of patients suffering from PPF were compared with those of controls, undergoing aseptic revision for other causes and primary THA for OA.

We demonstrated that 45% of the patients with PPF fulfilled the criteria of osteoporosis according to DXA measurements. Consistently, the mean BMD T-score was significantly lower than in both control groups consisting of patients undergoing aseptic revision or primary THA. With around 75% of cases being low-trauma fractures [2], PPF have a high rate of treatment-failure and mortality [15]. Demographic data suggest that most of these fractures may be related to osteoporosis. A recent study has shown that osteoporosis was present in 67 to 78% of patients with periprosthetic fractures, including but not limited to periprosthetic femoral hip fractures. The prevalence of osteoporosis varied depending on the diagnostic criteria used, including whether the diagnosis was made through clinical assessment and medical history or by using DXA or computed tomography data of the lumbar spine [16]. In another previous study, low BMD, defined as T-score ≤ − 1.0, was associated with a higher rate of intraoperative PPF compared to patients with normal BMD [17]. However, only twelve fractures were observed in total in this previous study, thus limiting the impact and generalizability of this finding. Indirect evidence supporting the potential osteoporosis-related nature of PPF was demonstrated by the fact that prior fragility fractures were shown to be a significant risk factor for PPF [18]. While other secondary causes may also cause PPF, for instance, aseptic or septic loosening with periprosthetic osteolysis (as defined per our exclusion criteria), osteoporosis has previously also been found to lead to a significantly higher number of medical, surgical, and overall complications in patients with PPF [19]. The collective evidence suggests that poor bone quality plays a major role with respect to the occurrence of PPF, but also in relation to poor outcomes and complications, which underlines the clinical importance of bone health assessment in hip arthroplasty.

High-risk patients should receive DXA prior to primary THA to allow appropriate treatment initiation [20]. In addition, repeated BMD assessments are also crucial as PPF can virtually occur at any time, with peaks in occurrence intraoperatively and early postoperatively [7, 8], but eventually also increasing over time [21, 22]. The guidelines of the National Osteoporosis Foundation recommend the fracture risk assessment tool (FRAX) for preoperative screening [23]. Studies investigating the effectiveness of this tool suggest that it has the potential to assess PPF risk and should ideally be calculated before and after THA [24]. However, there is a lack of implementation in everyday clinical practice. This is underlined by prior research, indicating that 75–80% of patients did not receive preoperative screening [10, 14], and of those at high risk of osteoporosis, around 10% received a DXA scan [5, 25]. Notably, there are currently no standardized definitions for identifying patients at high risk of osteoporosis in the context of arthroplasty. Therefore, clinicians typically rely on national guidelines and recommendations to define high-risk patients in general. In Germany, the national guideline defines a number of risk constellations in which screening for osteoporosis (primarily using DXA) should be initiated [12]. These include certain risk constellations in women aged 50 and over and in men aged 60 and over, including previous fragility fractures, rheumatoid arthritis, diabetes mellitus, neurological diseases, a history of proximal femur fracture in either parent, depression, heart failure, use of glucocorticoids > 2.5 mg, opioids, and many more. Furthermore, osteoporosis screening is recommended for women aged 70 and over and men aged 80 and over, regardless of any additional risk factors. However, as PPF may differ in their development from typical fragility fractures, one aim of future research is to determine individual risk constellations to identify high-risk patients who would benefit from DXA measurement prior to arthroplasty.

In addition to an adequate determination of BMD as a prerequisite for further preventive measures, the consequence of low BMD with regard to the surgical procedure, especially stem fixation, is a matter of ongoing debate. According to a previously conducted survey, over 60% of orthopedic surgeons reported that they would reconsider THA in cases of low BMD or would at least adapt the type of prosthesis fixation [26]. Furthermore, it is important to note that patients undergoing THA with uncemented stem fixation have a 14-and 10-times higher risk of intra- and postoperative PPF, respectively [21]. Based on these previous results, we hypothesized that patients with cemented stems would on average have to exhibit lower BMD values (i.e., more severe osteoporosis) to sustain a fracture. Indeed, when comparing DXA outcomes within our PPF cohort, we observed that patients with fractures around cemented stems showed a trend toward lower T-scores compared to uncemented stems, although this difference marginally failed to reach the statistical level of significance (*p* = 0.07). Additional age adjustment did not result in any significant differences in the corresponding Z-scores. Importantly, based on the aforementioned previous study [21] and further reinforced by our case study, cemented stem fixation should be considered for elderly patients and also in those with osteoporosis to minimize the risk of PPF.

Pharmacological treatment is the method of choice for improving BMD. More specifically, several studies have shown that periprosthetic BMD is improved by anti-resorptive drugs such as bisphosphonates or denosumab [27, 28], suggesting a reduction in PPF risk. Nevertheless, PPF risk reduction by bone-specific drugs has not yet been sufficiently investigated. Notably, the use of bisphosphonates, a first-line treatment for osteoporosis, before revision surgery was associated with an almost two-fold increase in implant survival time [29]. However, there are also contrasting observations that bisphosphonates may even increase the risk of intra- and postoperative PPF [30]. Nonetheless, the mentioned study did not account for osteoporosis severity as a confounding factor, which is why the diagnosis of osteoporosis rather than bisphosphonate treatment was most likely decisive for the increased rate of PPF [31].

A limitation of our study is that the number of included patients was rather small, which results in small sample sizes, especially for subgroup analyses within the PPF cohort. Additionally, the application of certain exclusion criteria may have impacted our ability to accurately determine the true prevalence of osteoporosis, potentially resulting in differences from what we have demonstrated. Nevertheless, this is the first study to compare gold standard-derived assessments of BMD using DXA measurements with adequately matched control groups in a cross-sectional study design. Other studies investigating the role of BMD on the occurrence of, for example, intraoperative PPF within a larger cohort of patients undergoing THA were ultimately limited by the occurrence of very few fractures [17]. A further limitation of our study is the inability of our study design to make predictive statements about DXA measurements in relation to fractures. However, it is noteworthy that our study is the first to adequately address and quantify the prevalence of osteoporosis within a PPF cohort. Therefore, there is a clear need for prospective studies to investigate the value of BMD measurements in the development of PPF. Finally, another limitation of our study is the absence of data on peripheral BMD, e.g., using forearm DXA or high-resolution peripheral quantitative computed tomography measurements. Further studies investigating the BMD at these sites and their predictive value on PPF should be conducted in the future.

In conclusion, patients who underwent revision surgery for PPF were significantly more likely to have osteoporosis compared to patients who underwent aseptic revision or primary THA. Consequently, it is likely that osteoporosis is a relevant risk factor for PPF that should be evaluated and treated to prevent and reduce the occurrence of this serious complication.

### Supplementary Information

Below is the link to the electronic supplementary material.

Supplementary file1 (DOCX 461 KB)

## Data Availability

The datasets used and/or analyzed during the current study are available from the corresponding author on reasonable request.

## Declarations

### Research Involving Human and Animal Participants

The study followed the rules of the Declaration of Helsinki and was approved by the local ethics committee (Hamburg Chamber of Physicians) under 2021-300036-WF.
