# Supplementary material for: Patients with Periprosthetic Femoral Hip Fractures are Commonly Classified as Having Osteoporosis Based on DXA Measurements
Source: Calcif Tissue Int. 2024 Jun 4;115(2):142–9. doi: 10.1007/s00223-024-01237-w (PMC11246254; doi:10.1007/s00223-024-01237-w)
Supplement: Supplementary file 1 — Supplementary file1 (DOCX 461 KB) [file 223_2024_1237_MOESM1_ESM.docx]

**Supplementary Material**

**Patients with periprosthetic femoral hip fractures are commonly classified as having osteoporosis based on DXA measurements**

**
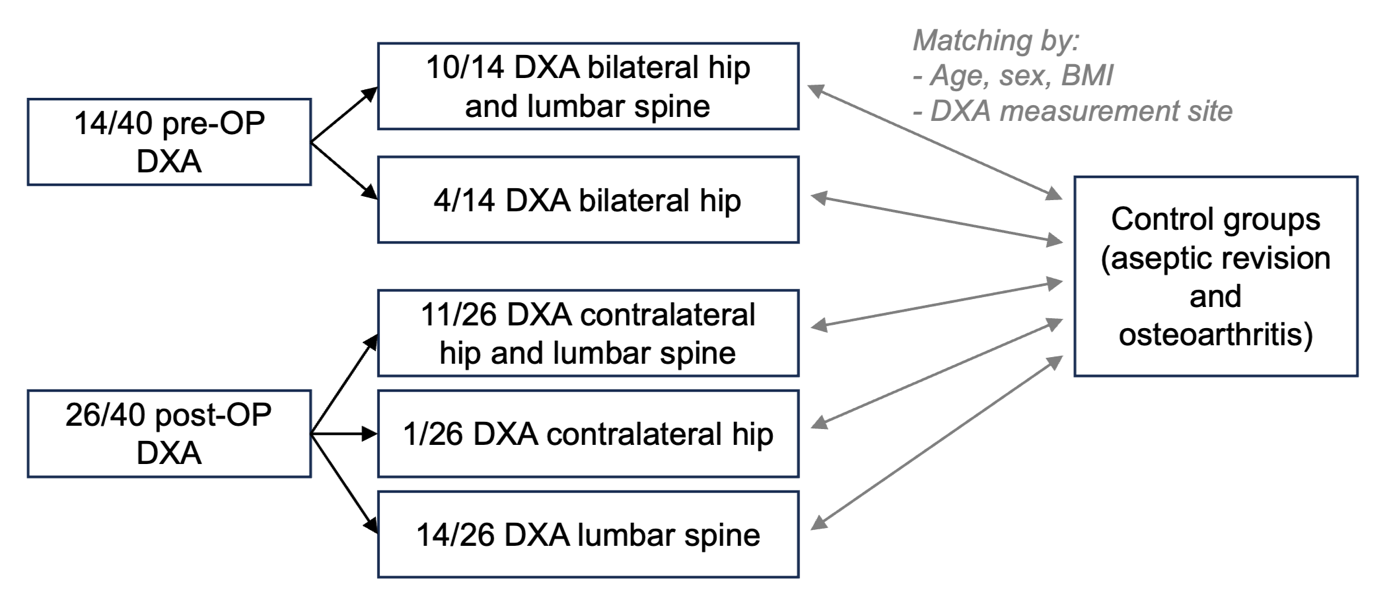
**

**Suppl. Fig. 1: Breakdown of the study cohort into preoperative vs. postoperative DXA measurements as well as the available measurement sites and comparison with the control groups.**

Abbreviations: DXA: dual-energy X-ray absorptiometry, BMI: body mass index.

**
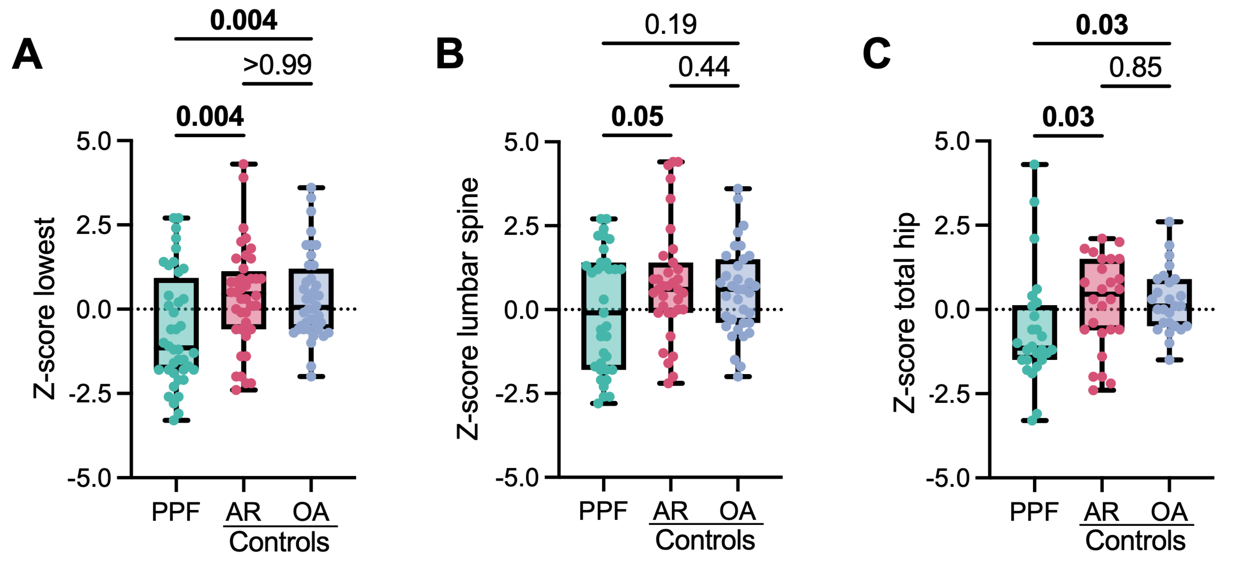
**

**Suppl. Fig. 2: Comparison of BMD Z-scores assessed by DXA between the periprosthetic fracture (PPF) cohort and both control cohorts.** Comparison of Z-scores when evaluating the (A) lowest Z-score of any measurement site, (B) lumbar spine, and (C) total hip. Bold indicates significant differences. Abbreviations: AR: aseptic revision, OA: osteoarthritis.

**
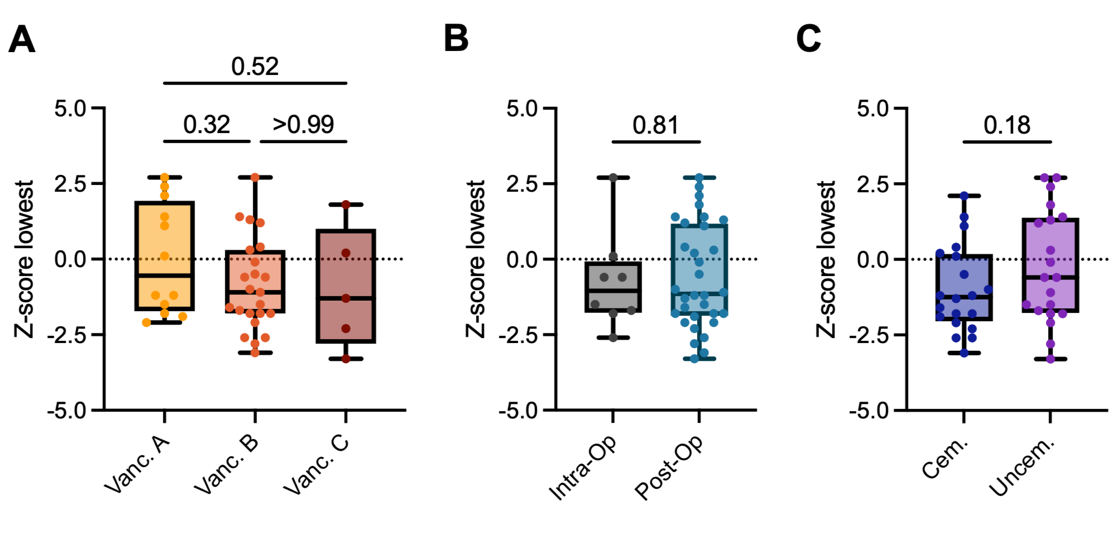
**

**Suppl. Fig. 3: Comparison of BMD Z-scores according to different clinical constellations.** (A) Comparison of Z-scores (lowest of any measurement site) between different types of PPF according to the Vancouver classification, (B) between intraoperative and postoperative fractures, and (C) between patients undergoing cemented vs. uncemented fixation.
